# Atypical Juvenile Pityriasis Rubra Pilaris: A Case Report of Early Onset With Late Diagnosis

**DOI:** 10.7759/cureus.30234

**Published:** 2022-10-12

**Authors:** Bakr A Albrakati, Ibrahim A Alshareef, Waseem K Alhawsawi, Khalid A Al Hawsawi

**Affiliations:** 1 Dermatology Department, Hera General Hospital, Makkah, SAU; 2 College of Medicine, King Saud Bin Abdulaziz University for Health Sciences, Jeddah, SAU; 3 Dermatology Department, King Fahad Hospital of the University, Al Khobar, SAU; 4 Dermatology Department, King Abdulaziz Hospital, Makkah, SAU

**Keywords:** pityriasis rubra pilaris, prp, papulosquamous, type v prp, atypical juvenile pityriasis rubra pilaris

## Abstract

Pityriasis rubra pilaris (PRP) is a rare inflammatory papulosquamous skin disease that has six distinct types. Type 5 PRP is called atypical juvenile PRP. Here we report the case of a 17-year-old boy with insignificant past medical history presenting with a history of persistent slowly progressing very itchy skin lesions since the age of seven years. The lesions were photoaggravated. No similar cases in the family were observed and the parents were not consanguint. Skin examination revealed scaly erythematous patches, papules and plaques all over his body. There were also ichthyosiform-like scales covering the whole body. Hair, nails, and mucus membranes examinations were normal. A 4-mm punch skin biopsy was taken. The dermis revealed hyperkeratosis with checkerboard pattern of orthokeratosis and parakeratosis, the granular layer was preserved and acanthosis with thick and short rete ridges. The dermis showed mild perivascular lymphocytic infiltrates. On the basis of the above clinicopathological findings, the diagnosis of pityriasis rubra pilaris (atypical juvenile type) (type 5) was made. The patient was started on isotretinoin capsule 20 mg twice a day and placed under periodic follow-up.

## Introduction

Pityriasis rubra pilaris (PRP) is a rare inflammatory papulosquamous skin disease of unknown etiology. The incidence was estimated to be one in 500,000 up to one in 5000 patients in India and the United Kingdom, respectively [1-3]. However, a higher incidence was reported within the pediatric group [4]. According to Griffiths’ classification, there are five distinct types of PRP. The sixth additional type was made by Miralles et al. which presents in HIV patients [3,5]. The majority of cases of PRP are sporadic, however, familial cases have been reported with type 5 [1,6]. The typical types (type 1 in adults and type 3 in children) are characterized by disseminated yellowish-pink scaly plaques surrounding islands of normal skin that show a cephalocaudal spread, and palmoplantar orange waxy keratoderma, whereas the atypical types (type 2 in adults and type 5 in children) are characterized by ichthyosiform scaling (more in type 5 than type 2); palmoplantar keratoderma with coarse and lamellated scales in type 2 and scleroderma-like changes of the fingers in type 5 [1]. The duration of the disease in types 2 and 5 is longer than in types 1 and 3 [1]. Follicular hyperkeratosis can be seen with any type. Type 5 is an atypical juvenile PRP that represents about 5% of PRP cases. It usually presents in the first decade of life [1]. Here we report a case of a 17-year-old male recently diagnosed with atypical juvenile PRP (type 5).

## Case presentation

**Figure 1:**
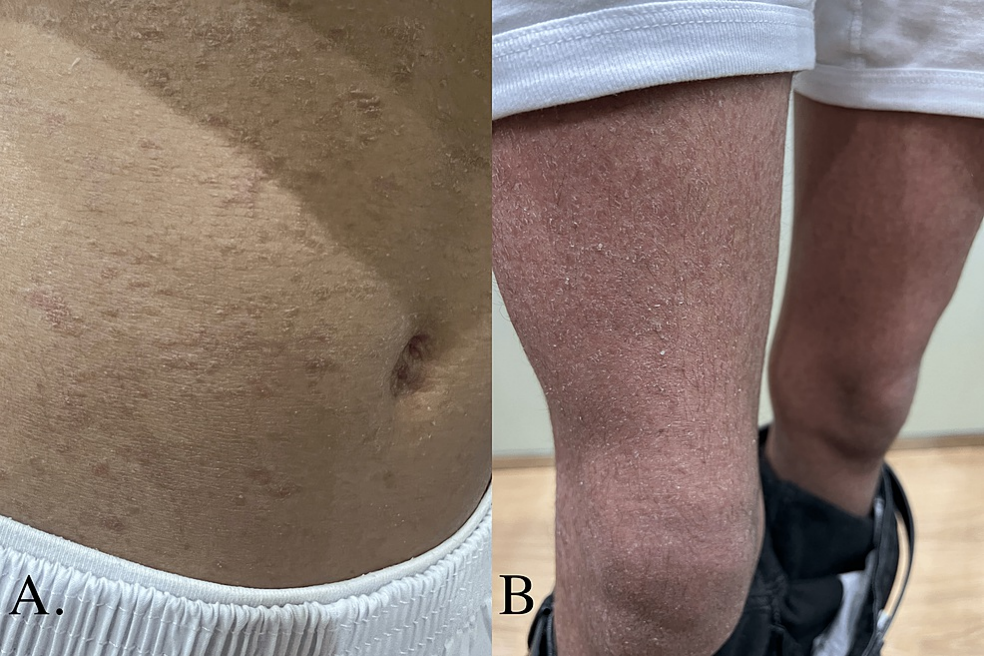
(A) Multiple scaly erythematous papules and plaques over the abdomen. (B) Ichthyosiform scales on the thighs

A 17-year-old boy with insignificant past medical history presented with a history of persistent very itchy skin lesions since he was seven years old. The lesions were persistent until now without history of remissions or exacerbations. The lesions started in small areas of the body that slowly progressed and spread to cover the whole body over two years. He has not been using any treatment except moisturizing cream. The lesions were photoaggravated. Review of systems was unremarkable. No similar cases were identified in the family and the parents were not consanguint. Skin examination revealed scaly erythematous patches, papules and plaques all over his body. There were also ichthyosiform-like scales covering the whole body (Figure 1).

**Figure 2:**
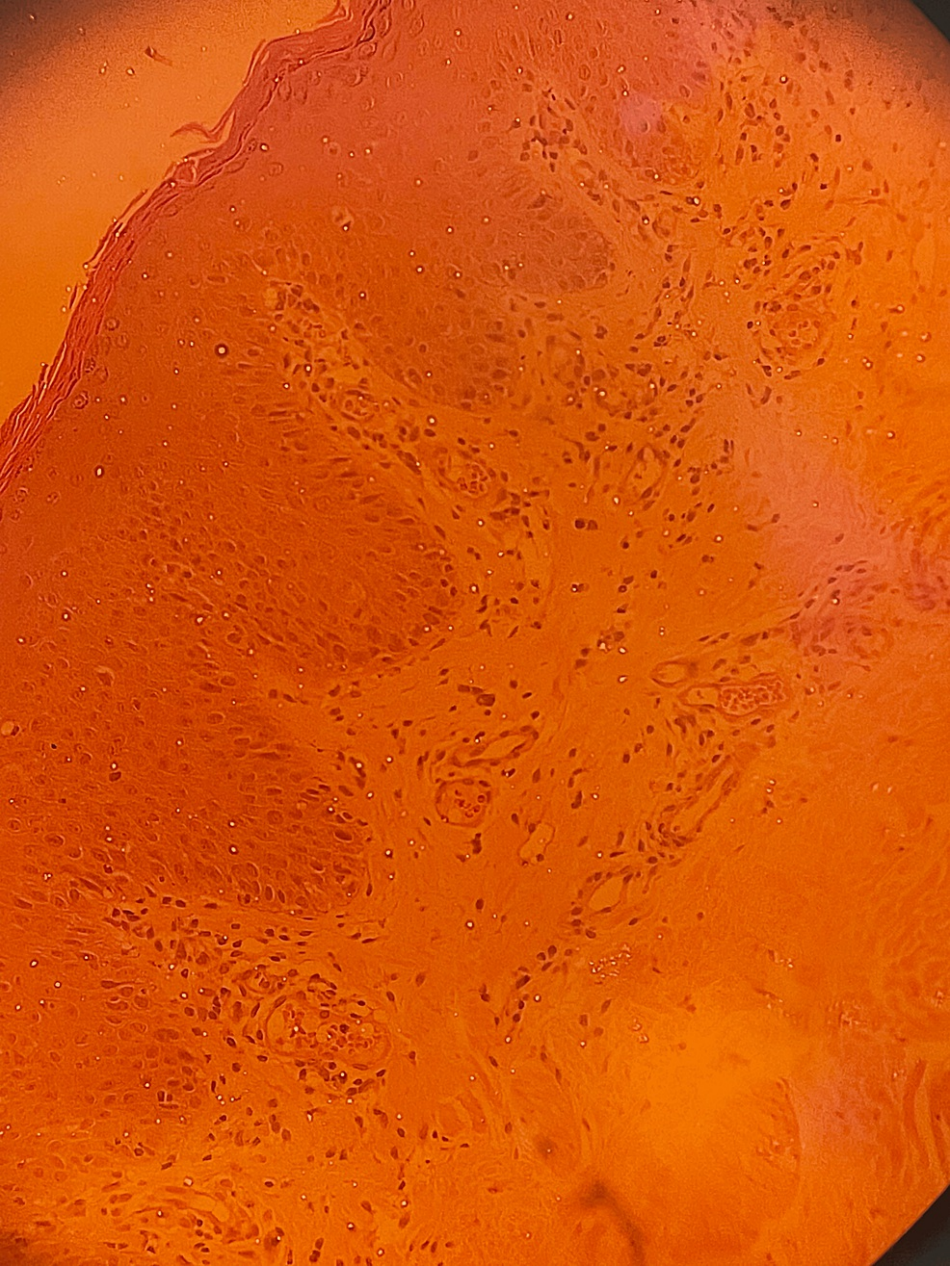
Skin biopsy results showing epidermal hyperkeratosis with checkerboard pattern of orthokeratosis and parakeratosis. Acanthosis with a thick and short rete ridges. The dermis showing mild perivascular lymphocytic infiltrate. hematoxylin & eosin stain; original magnification, x20

Hair, nails, and mucus membranes examinations were normal. The differential diagnosis included mycosis fungoides, pityriasis rubra pilaris, subcutaneous lupus erythematosus, ichthyosis and psoriasis. A 4-mm punch skin biopsy was taken. The dermis revealed hyperkeratosis with checkerboard pattern of orthokeratosis and parakeratosis, the granular layer was preserved and acanthosis with thick and short rete ridges. The dermis showed mild perivascular lymphocytic infiltrates (Figure 2).

On the basis of the above clinicopathological findings, the diagnosis of pityriasis rubra pilaris (atypical juvenile type) (type 5) was made. The patient was started on isotretinoin capsule 20 mg twice daily and put under periodic follow-up.

## Discussion

Pityriasis rubra pilaris (PRP) comprises six types. The skin lesions in all types are generalized except in type 4, the most common type of PRP in children, where the lesions are localized to elbow, knees, palms and knees. Generally, the most common type is type 1 [1]. Many cases cannot easily fit into any of these classifications. Patients may exhibit characteristics of one type that then evolve into another type. The onset of PRP may be acute appearing and spreading over a few days. However, the spread of lesions in our patient was gradual over years. The onset in our case was in the first few years of life. However, diagnosis was late due to difficulty of diagnosis and rarity of the disease. Most familial cases of PRP are type 5 which typically manifests as an autosomal dominant with a gain of function mutation on chromosome 17q25 in the caspase recruitment domain family 14 (CARD14) gene [6]. This gene is involved also in familial psoriasis vulgaris [7]. Interestingly, CARD14 was reported in familial as well as sporadic type 5 PRP [8]. Due to rarity of the disease, there are no randomized clinical trials on the treatment of PRP. Therefore, only retrospective case series and case reports - levels four and five of evidence - were the sources of recommendations. Oral retinoids (isotretinoin, acitretin, and alitretinoin) are considered the first-line agents for PRP. Thus we started our patient on isotretinoin 20 mg twice daily [9]. Adequate therapeutic trials of retinoids require at least four to six months. Phototherapy is one of the treatment modalities in PRP. However, it should be avoided in our patient since our he had a history of photoaggravation. Other systemic agents include methotrexate, biologics, TNF-α inhibitors, and Th17/IL-23 inhibitors, especially ustekinumab, cyclosporine, azathioprine, and apremilast [10,11]. Topical agents which include high-potency corticosteroids, tar, calcipotriene (calcipotriol), keratolytics, and tretinoin can be used as adjuncts to systemic therapy [12].

## Conclusions

PRP type 5 is considered one of the rarest diseases in dermatology that can be misdiagnosed for many years. Thus, a high index of suspicion through careful history and physical examination with clinicopathological correlation is needed. In addition, some individuals with PRP type 5 have familial forms including those with a CARD14 gene mutation. Therefore, genetic testing in patients with PRP type 5 is advised and family counselling is recommended.
